# A constrained-condylar fixed-bearing total knee arthroplasty is stabilised by the medial soft tissues

**DOI:** 10.1007/s00167-020-05995-6

**Published:** 2020-04-22

**Authors:** Kiron K. Athwal, Lukas Willinger, William Manning, David Deehan, Andrew A. Amis

**Affiliations:** 1 Department of Mechanical Engineering, Imperial College London, Exhibition Road, London, SW7 2AZ UK; 2 Department of Orthopaedic Surgery, Newcastle Freeman University Hospital, Newcastle, UK; 3 Musculoskeletal Surgery Group, Department of Surgery and Cancer, Imperial College London School of Medicine, London, UK

**Keywords:** Revision total knee arthroplasty, Stability, Medial collateral ligament, Semi-constrained implant, Total knee replacement, Constrained condylar prosthesis, Robotic testing

## Abstract

**Purpose:**

Revision constrained-condylar total knee arthroplasty (CCK-TKA) is often used to provide additional mechanical constraint after failure of a primary TKA. However, it is unknown how much this translates to a reliance on soft-tissue support. The aim of this study was therefore to compare the laxity of a native knee to the CCK-TKA implanted state and quantify how medial soft-tissues stabilise the knee following CCK-TKA.

**Methods:**

Ten intact cadaveric knees were tested in a robotic system at 0°, 30°, 60° and 90° flexion with ± 90  N anterior–posterior force, ± 8 Nm varus-valgus and ± 5 Nm internal–external torques. A fixed-bearing CCK-TKA was implanted and the laxity tests were repeated with the soft tissues intact and after sequential cutting. The deep and superficial medial collateral ligaments (dMCL, sMCL) and posteromedial capsule (PMC) were sequentially transected and the percentage contributions of each structure to restraining the applied loads were calculated.

**Results:**

Implanting a CCK-TKA did not alter anterior–posterior laxity from that of the original native knee, but it significantly decreased internal–external and varus-valgus rotational laxity (*p* < 0.05). Post CCK-TKA, the sMCL restrained 34% of the tibial displacing load in anterior drawer, 16% in internal rotation, 17% in external rotation and 53% in valgus, across the flexion angles tested. The dMCL restrained 11% of the valgus rotation moment.

**Conclusions:**

With a fully-competent sMCL in-vitro, a fixed-bearing CCK-TKA knee provided more rotational constraint than the native knee. The robotic test data showed that both the soft-tissues and the semi-constrained implant restrained rotational knee laxity. Therefore, in clinical practice, a fixed-bearing CCK-TKA knee could be indicated for use in a knee with lax, less-competent medial soft tissues.

**Level of evidence:**

Controlled laboratory study.

## Introduction

Condylar-constrained, non-linked total knee arthroplasty (CCK-TKAs) are designed to provide more inherent mechanical stability than standard primary implants, often by using larger tibial posts or having a more conforming fit between the femoral condyles and the tibial bearing surface [5]. There may be a perceived trade-off with these highly conforming designs: they could rely less on soft-tissue contribution which in turn can be easier to balance the knee in flexion and extension, however, overly restricting the laxity of the joint could increase stresses on the implant-bone interface [14]. Newer designs of CCK-TKA have been designed with features such as a gradually decreasing femoral radius, with the aim to maintain a consistent engagement with soft-tissue through the flexion arc whilst providing enough inherent stability in difficult revision cases [9, 10]. However, it is still quantitatively unknown how much this translates to a reliance on soft-tissue support such as the medial collateral ligament (MCL) for stability, which is a primary restraint to valgus laxity when intact with a primary TKA [3] but possibly compromised when the knee requires revision.

Therefore, the first aim of this study was to evaluate the laxity and stability of a modified design of CCK-TKA which is intended to engage the soft tissues across the arc of knee flexion–extension, and to compare it to the native knee. The second aim was to measure the contributions of the soft-tissues on the medial side of the knee to restraining the laxity of the CCK-TKA. The purpose was to provide data which inform the clinical evaluation of the knee and the safe zone of use at the time of revision and thus guide the choice of implant type. There is a lack of such objective data available, leaving the surgeon to make a subjective judgement. The null hypotheses were that the CCK-TKA with intact medial soft tissues would not have different laxity than the native knee, and that the superficial MCL (sMCL) would not be a major restraint in the CCK-TKA knee.

## Materials and methods

### Specimen preparation

Ten left-sided fresh-frozen cadaveric knees (mean age 71 ± 6 years, five males and five females) were obtained from a US-based tissue bank with Ethical approval from a local Human Tissue Authority Committee. The exclusion criteria of the specimens were intended to avoid abnormalities which would affect the behaviour of the intact ‘normal’ knee, which would act as the datum for later stages of the experiment; these criteria were assessed subjectively by the surgeon after each specimen had thawed, and included evidence of fixed flexion (that is: inability to extend the knee until the shafts of femur and tibia were parallel in the sagittal plane, misalignment of the femoral or tibial shafts in the coronal plane (This could not be measured meaningfully in the short specimens supplied, but was judged subjectively to appear ‘normal’), evidence of prior injury and/or surgery of the knee (by visual search for scars, medical summary notes supplied by the tissue bank, and lack of bone shape abnormality that might indicate an old healed fracture at the knee), osteophytes large enough to affect the adjacent soft tissues (that is: protruding so that they would deflect/tension the overlying soft tissues), ligament defects that would affect the laxity behaviour of the intact knee (checked via the normal clinical stability tests), or chondral defects that would alter the kinematics and ligament tension/slackness (surface roughness was accepted, but loss of cartilage volume would not be). The intracapsular factors were re-examined when the knee was opened for surgery. It was anticipated that one or two specimens would be rejected due to tissue damage or other technical errors, leading to eight for analysis. However, none of the ten knees examined had arthritic erosions or deformity that would affect their ‘intact’ behaviour, so all were used in the study, and none were eliminated because of technical problems.

The distal tibia/ fibula and the proximal femur were cut to lengths of 160 and 170 mm from the joint line respectively to allow consistent mounting geometry in the robot. The fibula was fixed to the tibia in its anatomical position using a tricortical bone screw. All skin and tissue were maintained on the tibia and femur between 105 and 110 mm from the joint line respectively; distal to this on the tibia and proximal on the femur were skeletonised and fixed in 60 mm diameter cylindrical steel pots using polymethyl-methacrylate (PMMA) bone cement (Simplex, Kemdent, UK). A midline incision to the skin and subcutaneous layer followed by a medial parapatellar arthrotomy was performed to access the joint space, and a jig with a pointer was used to locate the midpoint between the tips of the tibial spines and align this with the axis of the bone pot [1]. The femur was cemented in the bone pot while mounted in the robot in full extension with the posterior condylar axis aligned parallel to the femoral fixture.

### Native knee kinematics

The native knee was manually flexed 20 times to minimise soft-tissue hysteresis, and then tested in a robotic testing system previously described [3, 4]. The femur was fixed rigidly to the base of the robot and the tibia to a 6 degree-of-freedom (DOF) force + moment sensor on the end-effector of the manipulator. The knee was flexed in the robot from full extension to 90° while the system minimised the forces and moments in the other five DOF. The positions of the knee at full extension, 30°, 60° and 90° flexion were recorded and used as starting positions for the following tests. Loads imposed on the tibia were: ± 90  N anterior–posterior (AP) force, ± 5 Nm internal–external rotation (IE) torque and ± 8 Nm varus-valgus (VV) moment. In each test, the robotic system applied the load in the primary DOF, maintained the flexion/ extension DOF and minimised the loads in the other four DOF.

### TKA implantation and soft-tissue release

**Fig. 1:**
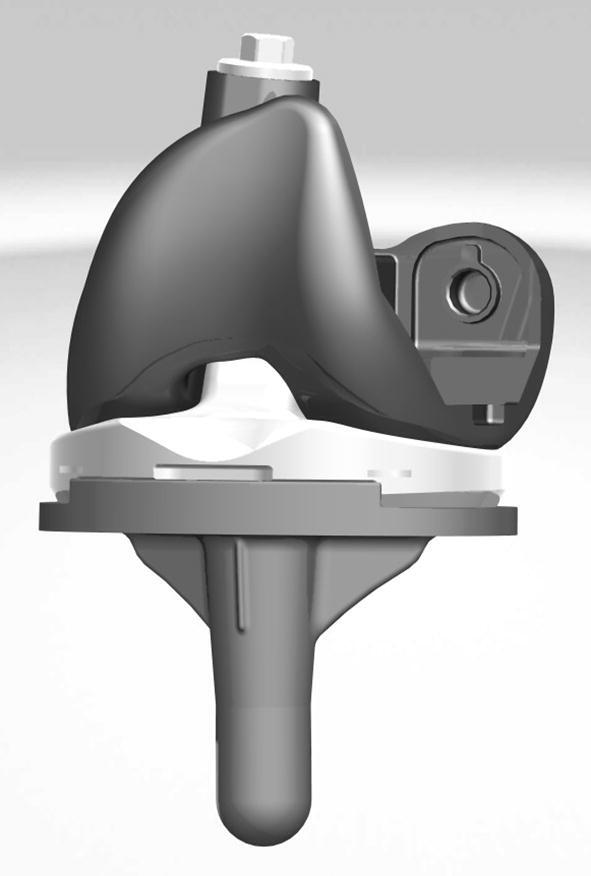
The Attune semi-constrained revision TKR with high-conforming fixed bearing. The tests were performed without stem extensions in the intact cadaveric bones

After collecting native knee data, the specimen was removed from the robot and a fixed-bearing CCK-TKA without intramedullary stem extensions (Fig. 1) was implanted by a post CCST knee fellowship trained orthopaedic surgeon as per implant manufacturer recommendation (Attune Intuition instruments surgical technique, DePuy Synthes, and Attune revision knee system fixed bearing surgical technique, DePuy Synthes) using a medial parapatellar approach and a measured resection technique. It was confirmed there was no evidence of significant OA (that is: localised surface damage, but no obvious erosions) and therefore no secondary soft tissue or posterior capsular contractions and no need to remove any osteophytes. The femur was prepared using an intramedullary guide and 9 mm distal femoral resection set at 5° valgus and cut with a 1.2 mm thick saw blade; there was no evidence during the surgery that this fixed angle clashed with any abnormal valgus angulation in any specimen, between the joint and the shaft of the femur. Femoral sizing was undertaken using anterior referencing, with the transepicondylar axis and Whiteside’s line to reference femoral component rotation. The tibia was prepared with a 10 mm deep cut from the lateral tibial plateau. A 3° posterior slope cutting block was used and care was taken to ensure that the dMCL was not compromised. The alignment of the tibial cut was ensured by placing a guide rod along the anterior tibial spine of the short specimen. Trial femoral components and tibial baseplates and bearings were inserted to ensure patellar tracking was satisfactory. No abnormal medial or lateral joint space opening occurred when a varus-valgus load was applied by the surgeon (that is, a judgement by the surgeon that the varus-valgus laxity was restored to normal behaviour, allowing 1–3 mm joint space opening at both medial and lateral aspects, as per the surgical technique), and to check that the 6 mm polyethylene insert thickness ensured equal flexion and extension gaps. The 9 mm femoral resection and the 10 mm tibial resection matched the thicknesses of the prosthetic components with the 6 mm tibial insert; the Attune fixed-bearing revision inserts are the same articular bearing geometry and thickness as the PS knee inserts. No soft-tissue releases were performed. Both tibial and femoral components were used without stem augments and were cemented to the bone using PMMA. Intramedullary stems were not required because of secure fixation as for a primary TKA and in anticipation of relatively low loads when testing. In all knees, a satisfactory clinical balance was achieved in the coronal, sagittal and axial planes throughout the arc of flexion, and it was confirmed that a full range of flexion–extension was achieved. Thus, these knees were defined by the surgeon as not being overconstrained by the implantation technique.

The CCK-TKA knee was fixed back to the robot and a new path of passive flexion from full extension to 90° was performed, after which the same set of loading conditions was applied: ± 90  N AP, ± 5 Nm IE and ± 8 Nm VV applied at the new full extension, 30°, 60° and 90° positions.

Whilst the CCK-TKA knee remained attached to the robot, the deep MCL (dMCL), sMCL and posteromedial capsule (PMC) were sequentially released. The dMCL was transected using a scalpel deep to the ligament and just distal to the tibial plateau. The anterior fibres of the sMCL were released from their distal tibial attachment subperiosteally using an osteotome [19], and then the rest of the sMCL was transected at the joint line. Finally, the PMC fibres from the posterior border of the dMCL across to the semimembranosus tendon at the posteromedial corner of the tibial plateau were transected with a scalpel. After each stage (the sMCL being released in two stages), the robot replayed the kinematics from the intact CCK-TKA stage. Therefore, using the principle of superposition [16], any decrease in force/moment at each stage related to the restraint provided by that transected structure.

### Statistical analysis

A custom Matlab (Mathworks, Natick, MA) script was used to calculate the mean peak forces/torques, translations/ rotations, and soft-tissue contributions as a percentage drop in force/torque from the original values. The following statistical analyses were performed using SPSS (IBM SPSS Statistics, version 24, Armonk, NY):

1. Two-way repeated-measures analysis of variance (RM-ANOVA) was performed to compare native and CCK-TKA laxities across different flexion angles.
2. One-way RM-ANOVA at each flexion angle was performed to compare force/torque contributions of the different soft-tissues.

When differences were found, post hoc t tests with Bonferroni correction were performed with significance level set at *p* < 0.05. A power calculation, based on a mean change in translation of 3.5 ± 3 mm and rotation of 3.7 ± 3.2° in a prior study [3], determined that a sample size of eight would be needed to detect a significant laxity change and soft-tissue contributions of 9% with 80% power and 95% confidence. Therefore, a significant restraint for a given flexion angle was defined as having a statistically significant mean resisting contribution greater than a threshold value of 10%.with *p* < 0.05 [2–4].

## Results

### Laxity of the native and replaced tibiofemoral joint

#### Anterior–posterior translation laxity

**Fig. 2:**
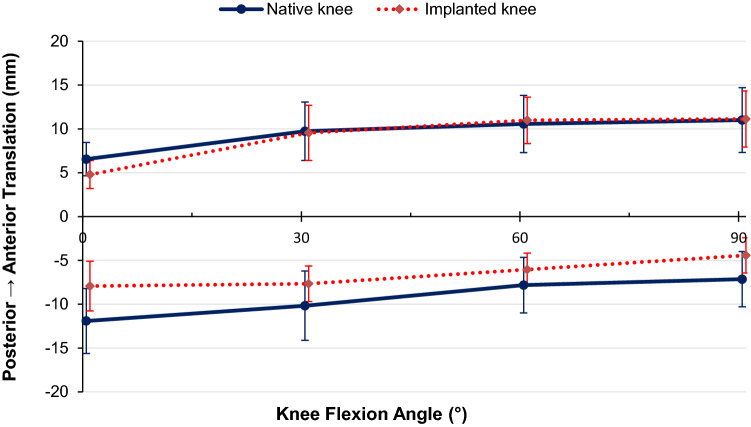
Anterior (positive) and posterior (negative) tibiofemoral translation of the native knee and CCK-TKA implanted knee in response to ± 90  N anterior–posterior force (mean ± standard deviation, *n* = 10)

Under a 90  N anterior or posterior force, no significant difference was found between the native knee laxity and the replaced knee laxity (Fig. 2).

### Internal–external rotation laxity

**Fig. 3:**
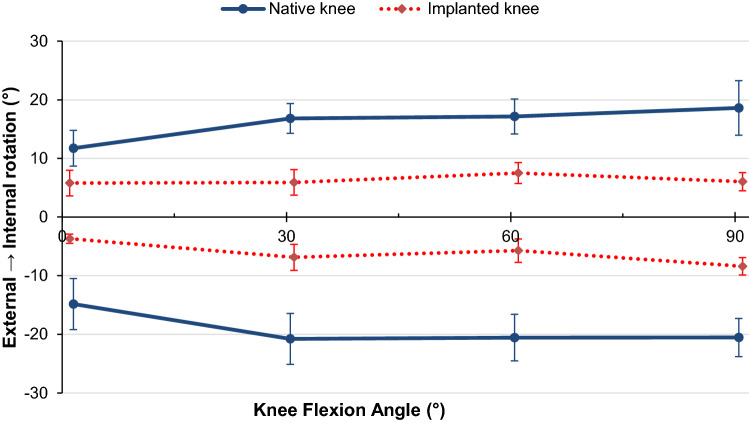
Internal (positive) and external (negative) tibial rotation of the native knee and CCK-TKA implanted knee in response to ± 5 Nm internal–external torque (mean ± standard deviation, *n* = 10)

When a 5 Nm internal rotation torque was applied, the fixed-bearing CCK-TKA significantly decreased internal rotational laxity with respect to the native knee state by an average of 10° across all tested flexion angles (*p* < 0.001, Fig. 3).

In response to 5 Nm external rotation torque, the fixed-bearing CCK-TKA significantly decreased external rotational laxity with respect to the native knee state by an average of 13° across flexion (*p* < 0.001 at all angles, Fig. 3).

### Varus-valgus angulation laxity

**Fig. 4:**
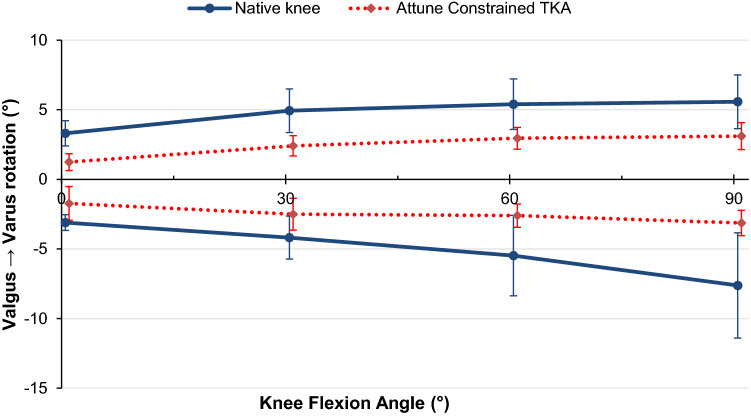
Varus (positive) and valgus (negative) angulation of the native knee and CCK-TKA implanted knee in response to ± 8 Nm varus-valgus moment (mean ± standard deviation, *n* = 10)

Under an 8 Nm varus moment, the CCK-TKA significantly decreased varus angulation laxity with respect to the native knee state by an average of 2° across flexion (*p* < 0.001, Fig. 4).

When an 8 Nm valgus moment was applied, the CCK-TKA restricted valgus angulation laxity by an average 3° across flexion compared to the native knee (Fig. 4, *p* = 0.013 at full extension, *p* = 0.012 at 30° and 60°, *p* = 0.006 at 90°).

## Restraint of replaced tibiofemoral joint laxities

### Restraint of anterior–posterior translation

**Fig. 5:**
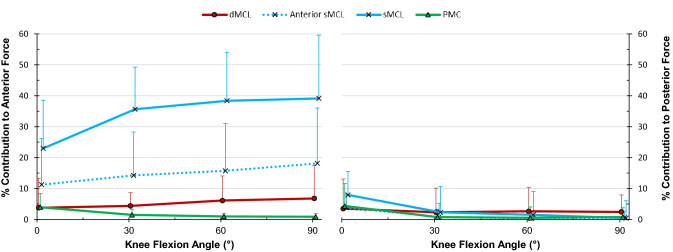
Percentage contributions of the deep medial collateral ligament (dMCL), superficial MCL (sMCL), anterior fibres of the sMCL and posteromedial capsule (PMC) in resisting 90  N anterior (left) and posterior (right) forces in the CCK-TKA implanted knee (mean ± standard deviation, *n* = 10)

In the CCK-implanted knee, the entire sMCL restrained an average of 34% of the anterior translation force across full extension to 90° flexion (Fig. 5, *p* < 0.001). Release of just the anterior sMCL fibres using an osteotome [19] showed that they had restrained 11% of the anterior translation force at full extension (*p* = 0.003), up to 18% at 90° (*p* = 0.025).

None of the soft-tissues tested restrained more than 10% of the posterior drawer force (Fig. 5).

### Restraint of internal–external rotation

**Fig. 6:**
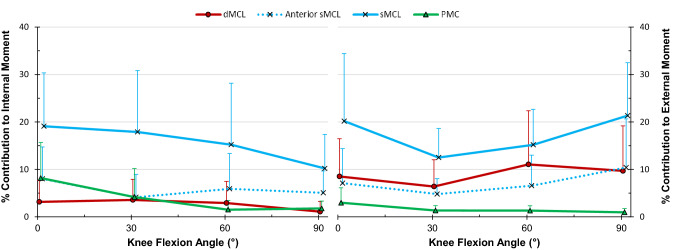
Percentage contributions of the deep medial collateral ligament (dMCL), superficial MCL (sMCL), anterior fibres of the sMCL and posteromedial capsule (PMC) in resisting 5 Nm internal (left) and external (right) tibial rotation torque in the CCK-TKA implanted knee (mean ± standard deviation, *n* = 10)

The sMCL contributed an average of 16% of the restraint of internal rotation across flexion (Fig. 6, *p* = 0.004 at full extension—*p* = 0.011 at 90°).

The sMCL provided an average of 17% of the restraint of external rotation (Fig. 6, *p* = 0.011 at full extension—*p* = 0.002 at 90°). The dMCL resisted approximately 10% of the external rotation torque (*p* = 0.019 and 0.016 at 60° and 90° respectively).

### Restraint of varus-valgus angulation

**Fig. 7:**
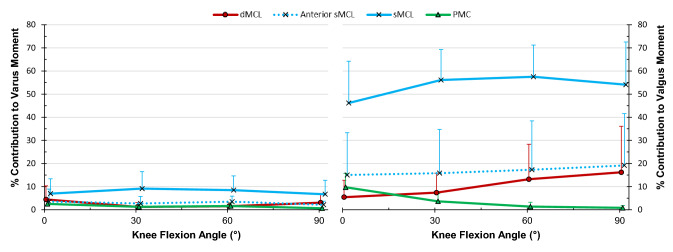
Percentage contributions of the deep medial collateral ligament (dMCL), superficial MCL (sMCL), anterior fibres of the sMCL and posteromedial capsule (PMC) in resisting 8 Nm varus (left) and valgus (right) moment in the CCK-TKA implanted knee (mean ± standard deviation, *n* = 10)

None of the medial soft-tissues were significant restraints to varus angulation, as expected (Fig. 7).

The sMCL provided an average of 53% of the restraint to valgus (Fig. 7, *p* < 0.001 at all flexion angles). The anterior fibres of the sMCL resisted 15–19% of the valgus restraint across full extension to 90° flexion (*p* = 0.034 at 90°), and the dMCL resisted more than 10% of the moment at 60° and 90° flexion (*p* = 0.031 and 0.040 respectively).

## Discussion

The most important finding of this study of a fixed-bearing CCK-TKA is that the sMCL provides an important restraint to valgus, internal–external rotations and anterior drawer in addition to that provided by the prosthesis. The fixed-bearing CCK-TKA with a fully competent sMCL was also found to constrain internal–external rotation and varus-valgus angulations more than the native knee, so the null hypotheses could be rejected. The high percentage of restraint provided by the combination of the TKA mechanism plus the intact soft tissues, which led to overconstraint of the joint below normal laxity, shows clearly that the CCK-TKA is not indicated when the medial ligaments are intact and their restraint is adding to the restraint provided by the engagement of the prosthetic tibial post within the femoral component box/cam space. At the other extreme, MCL insufficiency is usually an indication for a more-constrained rotating-hinge TKA rather than rely on the polyethylene post of the CCK to resist the loads. These findings suggest that the CCK-TKA may be more suited in a knee with a lax, less-competent sMCL, when the restraining loads will be shared between the soft tissues and the prosthesis as the laxity relaxes towards normal; the intent of a semi-constrained CCK-TKA is that it could provide stability in case of soft tissue laxity, but not to compensate for the absence of soft-tissue restraint, and excessive load on the MCL may cause pain.

When the knee was loaded with an 8Nm valgus moment the medial soft-tissues (dMCL, sMCL and PMC) restrained an average of 68% of the moment, across full extension to 90° flexion. A previous study investigating the same soft-tissues with an older design of constrained condylar implant (DePuy Synthes Rotating-Platform TC3) and the same loading conditions found an average of 72% across flexion angles (*p* = 0.138 by unpaired 2-way *t* test, n.s.) [2]. There were similar findings under ± 50  N AP loads: with anterior drawer the medial soft-tissues resisted 51% for both the Attune and the TC3, and with posterior drawer 17% and 21% with the Attune and TC3 respectively (*p* = 0.140, n.s.). This shows that, despite comparing a fixed-bearing CCK-TKA in the current study to a rotating-bearing CCK-TKA, both design types utilise proportions of support from soft-tissue that are not significantly different.

A cadaveric study comparing Attune Revision and Sigma TC3 (both fixed-bearing) under ± 6 Nm VV and ± 4 Nm IE torques found less VV motion in the Attune than the TC3 in mid-flexion and similar IE rotation between the two implants [11]. The Attune IE and VV laxity patterns were found to be very consistent with our present study, which suggests that regardless of experimental set-up and variation in cadaveric specimens the fixed-bearing Attune provides a consistent constraint to rotation.

The implications of these kinematic laxity data and of the constraint provided by the CCK-TKA implants on load transfer and resulting fixation are worth further investigation. Semi-constrained TKA constructs have previously been found to reduce the laxity of posterolateral corner deficient knees compared to primary posterior-stabilised TKA, in part due to the larger posts and greater tibial insert concavity taking a greater stabilising role [12]. However, the widely accepted opinion was that increasing the amount of inherent constraint in an implant increases the stresses at the bone-implant interface which in turn may lead to early loosening [14]. This effect may be countered by increasing the engagement of the components with the bone, by means such as the addition of metaphyseal sleeves or intramedullary stems [6, 7]. However, a larger implant requires more destructive loss of bone, which then leaves even more difficult surgery for any further required revisions [5].

Recent finite element studies have predicted micromotion under various joint-loading scenarios at the tibial tray/bone interface of Attune CCK-TKAs to be comparable to primary implants [6, 7]. However Samiezadeh et al. found that in full extension, the stresses on the bone-implant interface of a different constrained condylar design were increased when the MCL was completely deficient [17]. This raises the question of what level of constraint is needed to provide stability with an absent or lax MCL, yet also to avoid early fixation failure. Gustke suggested, using physical varus/valgus stress examinations and radiographs, that in the case of an absent MCL a rotating-hinged implant should be used unless the MCL can be reconstructed [13]. Sculco also advised using a rotating-hinged implant if the MCL is deficient, but made the distinction that the CCK-TKA could be used in knees with a present but lax MCL [18].

This study suggests that a balance should be achieved with the CCK-TKA: even a partly-released/deficient sMCL played an important role in stability, so this CCK-TKA could be considered with some MCL deficiency. However, this study has only considered soft tissue constraints, and other factors such as the possibility of wear of the polyethylene post or the bone resection and fixation must also be considered. This study found that if the MCL is fully competent and the fixed-bearing CCK-TKA is implanted, the resulting knee construct is more constrained in rotational planes than the native knee. One explanation for this could be that the knees were overstuffed, tensing the soft tissues, but the implantation method and evaluation of the resulting construct was exactly as used routinely in the authors’ clinical work, and the knees were confirmed to retain their full range of flexion–extension. Some surgeons may choose to excise a greater thickness of bone than that of the prosthetic components to ensure that overconstraint does not occur, because arthritic knees may have soft tissue stiffness that was not present in the specimens used in the present study. However, the explanation for the rotational overconstraint in the present work is that the internal–external and varus-valgus rotations were restrained by a combination of soft-tissue tensions plus post-box-cam engagement. For example, at 30^o^ flexion, the native knee rotational laxity was 17° IR–21^o^ ER, post CCK-TKA it was 6° IR–7° ER, and the implant itself allowed approximately 5° IR–5° ER when post/box engagement occurred. It appears to be inevitable that a fixed-bearing CCK-TKA which allows 10° IE rotation must lead to overconstraint, when the native knee had 38° range, and therefore that the IE overconstraint reported in this work was not a result of surgical factors.

The dMCL provided significant restraint of both tibial external rotation and valgus angulation. This shows that the tibial tray insertion had not damaged the attachment of the dMCL to the medial rim of the tibial plateau. These roles for the dMCL have been shown in both the native [15] and TKA knee [8], and imply the desirability of preserving this structure.

Limitations of this study include the time-zero nature of in-vitro studies which cannot account for healing of soft-tissues back onto the bone. Additionally, the testing protocol is not indicative of the dynamic loads experienced during the gait cycle and therefore care must be taken when applied to post-operative conditions. However, the applied forces accurately model joint laxity evaluation of a supine patient with relaxed muscles, and therefore gives a good representation of the decisions faced by a surgeon during time of operation. The realism of the work was limited by having to use normal or near-normal (non-arthritic) knees, which are what are available from tissue banks, when studying the behaviour of a prosthesis intended usually for use in revision surgery. As such, the resulting data may be viewed as being ideal knee stability behaviour for the implant design; further work might explore development of a bone and/or soft tissue deficient knee model. The cadaveric specimens used were short to reduce bending loads when tested by the robot. In addition, the loads imposed represented clinical manual examination. Therefore long intramedullary fixation stems were not needed, which differs from the clinical situation. While that would not have affected the prosthetic joint mechanics, it may have influenced the alignment and hence also affected the kinematics. There was no evidence that that occurred in this experiment, as seen by the central zones of laxity of the replaced knee, within the ranges of native laxity (Figs. 3 and 4).

This study shows that the constrained condylar fixed-bearing CCK-TKA provides an inherent constraint for a stable knee joint under per-operative investigation. Further studies with full joint contact forces and muscle loads are required to help surgeons decide the correct implant for a patient with ligamentous deficiency after a failed primary implant and ensure that the stresses are not such that early loosening is a large risk.

## Conclusion

This study has quantified the relative contributions of the medial soft-tissues to the stability of a fixed-bearing CCK-TKA and confirmed that the sMCL is a significant restraint to valgus, internal–external rotations and anterior drawer if the MCL is intact in the presence of this ‘semi-constrained’ CCK-TKA device. The fixed-bearing CCK-TKA device led to over-constraint of the knee joint in rotation when the MCL was intact, so the CCK-TKA may be more suited in a knee with a lax, less-competent sMCL.
